# Long-term adverse outcomes in survivors of childhood bone sarcoma: the British Childhood Cancer Survivor Study

**DOI:** 10.1038/bjc.2015.159

**Published:** 2015-05-19

**Authors:** M M Fidler, C Frobisher, J Guha, K Wong, J Kelly, D L Winter, E Sugden, R Duncan, J Whelan, R C Reulen, M M Hawkins

**Affiliations:** 1 Centre for Childhood Cancer Survivor Studies, School of Health and Population Sciences, Public Health Building, University of Birmingham, Birmingham B15 2TT, UK; 2 Orthopaedic Department, Royal Hospital for Sick Children, Yorkhill, Glasgow G3 8SJ, UK; 3 NIHR University College London Hospitals Biomedical Research Centre, London NW1 2PG, UK

**Keywords:** childhood cancer, bone sarcoma, long-term outcomes, Ewing sarcoma, osteosarcoma, bone cancer, paediatric cancer, late effects

## Abstract

**Background:**

With improved survival, more bone sarcoma survivors are approaching middle age making it crucial to investigate the late effects of their cancer and its treatment. We investigated the long-term risks of adverse outcomes among 5-year bone sarcoma survivors within the British Childhood Cancer Survivor Study.

**Methods:**

Cause-specific mortality and risk of subsequent primary neoplasms (SPNs) were investigated for 664 bone sarcoma survivors. Use of health services, health and marital status, alcohol and smoking habits, and educational qualifications were investigated for survivors who completed a questionnaire.

**Results:**

Survivors were seven times more likely to experience all-cause mortality than expected, and there were substantial differences in risk depending on tumour type. Beyond 25 years follow-up the risk of dying from all-causes was comparable to the general population. This is in contrast to dying before 25 years where the risk was 12.7-fold that expected. Survivors were also four times more likely to develop a SPN than expected, where the excess was restricted to 5–24 years post diagnosis. Increased health-care usage and poor health status were also found. Nonetheless, for some psychosocial outcomes survivors were better off than expected.

**Conclusions:**

Up to 25 years after 5-year survival, bone sarcoma survivors are at substantial risk of death and SPNs, but this is greatly reduced thereafter. As 95% of all excess deaths before 25 years follow-up were due to recurrences and SPNs, increased monitoring of survivors could prevent mortality. Furthermore, bone and breast SPNs should be a particular concern. Since there are variations in the magnitude of excess risk depending on the specific adverse outcome under investigation and whether the survivors were initially diagnosed with osteosarcoma or Ewing sarcoma, risks need to be assessed in relation to these factors. These findings should provide useful evidence for risk stratification and updating clinical follow-up guidelines.

Primary malignant bone sarcomas account for 4.8% of all childhood cancers in the United Kingdom (Stiller, 2007). Approximately 65 cases occur each year, of which the principal tumour types are osteosarcoma (53%) and Ewing sarcoma (39%) (Stiller, 2007). Although the incidence is low, survival after bone sarcoma has increased substantially. Since the 1970s 5-year survival has risen from 23 to 64% mainly due to the introduction of modern chemotherapy (Stiller, 2007). Consequently, as the number of individuals treated for childhood bone sarcomas increases, it becomes even more important to investigate the risk of the long-term effects of this childhood cancer and its treatment.

This study assessed adverse outcomes among bone sarcoma survivors diagnosed between the ages of 0 and 14 years within the British Childhood Cancer Survivor Study (BCCSS). Key advantages of the BCCSS compared with other studies are that it is a large, population-based cohort with 30.4% of individuals diagnosed with bone sarcoma surviving to age 45 years at least. Therefore, adverse health and social outcomes beyond 35 years post diagnosis in these childhood cancer survivors can be examined much more satisfactorily than has been possible in previous smaller or non-population-based studies with limited follow-up (Gianinazzi *et al*, 2013; Jazbec *et al*, 2004; Cardous-Ubbink *et al*, 2007; Armstrong *et al*, 2009; Casagranda *et al*, 2013). In this study, we investigated the long-term risk of premature mortality, developing a subsequent primary neoplasm, health-care usage, health and marital status, alcohol and smoking habits, and educational attainment among 5-year childhood bone sarcoma survivors.

## Materials and methods

### British Childhood Cancer Survivor Study

The BCCSS, which has been described previously in detail (Hawkins *et al*, 2008), is a population-based cohort comprised of 17 980 individuals; it includes 664 bone sarcoma survivors diagnosed with cancer before the age of 15, between 1940 and 1991 in Great Britain, and who have survived at least 5 years. The cohort was ascertained through the National Registry of Childhood Tumours, which has a high estimated level of completeness (∼99%) (Kroll *et al*, 2011). Ethical approval for the study was obtained from a Multi-Centre Research Ethics Committee and every Local Research Ethics Committee in Britain.

When treatment exposures within this cohort were investigated across 5-year calendar year bands, we found that before 1976, where our radiotherapy and chemotherapy treatment completeness was 98.4% and 88.4%, respectively, the majority of bone sarcoma survivors received radiotherapy (76.3%), with only a small proportion receiving chemotherapy. A distinct change in treatment practice was then observed from 1976 onwards where broadly all survivors received chemotherapy and Ewing sarcoma survivors additionally received radiotherapy. Thus, in order to address the incompleteness of treatment information in more recent diagnosis years, which was due to decreasing availability of recorded radiotherapy and chemotherapy details at the National Registry of Childhood Tumours during this period, our analyses were undertaken for bone sarcoma survivors overall and separately for osteosarcoma and Ewing sarcoma, which serve as proxies for treatment exposures. Therefore, osteosarcoma survivors were likely to have received radiotherapy if diagnosed before 1976 and only chemotherapy if diagnosed from 1976 onwards, whereas all Ewing sarcoma survivors were likely to have received radiotherapy, with only those diagnosed after 1976 additionally receiving chemotherapy. Consequently, those surviving beyond 25 years from 5-year survival were likely to have only received radiotherapy, whilst those with <25 years follow-up were likely to have received only chemotherapy or chemotherapy and radiotherapy depending on tumour type.

### Record linkage ascertained outcomes

Deaths and subsequent primary neoplasms (SPNs) were ascertained for the entire BCCSS cohort through record linkage with the National Health Service Information Centre, which includes the population-based national death and cancer registries. This linkage ensures that the BCCSS is notified whenever a survivor has died or developed a SPN. To determine the expected number of deaths or incident cancers, person-years for each sex-specific, age-specific (5-year bands), and calendar year-specific (1-year bands) stratum were multiplied by the corresponding general population rates for specific cause(s) of death and incident cancers occurring throughout England and Wales.

#### Cause-specific mortality

For our mortality analysis, the death certificate and underlying cause of death, as coded by the Office for National Statistics using the relevant *International Classification of Disease,* were obtained. The underlying cause of death was then confirmed by a clinician using available medical records. Time at risk started at 5-year survival and continued until individuals exited from risk at the first occurrence of emigration, death, or 31 December 2010 which was the date of the most recent vital status update on the entire cohort from the National Health Service Information Centre. The standardised mortality ratio (SMR) was defined as the ratio of observed to expected number of deaths. The absolute excess risk (AER) was defined as the observed minus the expected number of deaths divided by person-years at risk multiplied by 10 000. Cumulative mortality for a specific cause of death was calculated by treating other causes of death as competing risks.

#### Subsequent primary neoplasms

Confirmation of all SPNs was undertaken by writing to the relevant clinician(s) to obtain diagnostic reports to confirm site, type and date of diagnosis. Time at risk for a SPN began at 5-year survival and individuals exited from risk at the first occurrence of an SPN, emigration, death, or 31 December 2006 which was the most recent date up to which all potential SPNs had been ascertained and validated. Standardised incidence ratios (SIRs) were calculated as the ratio of observed to expected number of neoplasms. The AERs were calculated as described previously for the mortality analyses. Cumulative incidence for the first occurrence of a SPN was computed treating death as a competing risk.

### Questionnaire ascertained outcomes

Health-care usage, health and marital status, alcohol and smoking habits, and educational attainment were obtained via the BCCSS questionnaire. To be eligible to receive the BCCSS questionnaire survivors in the cohort had to be alive and aged at least 16 years at questionnaire send-out (median year 2002). Of the 664 bone sarcoma survivors, 506 survivors met this eligibility criteria and were contacted; amongst survivors who were ineligible, the majority had died before the questionnaire send-out (*n*=106). Ultimately, 411 (81.2%) returned a completed questionnaire. All comparisons with the general population were adjusted for age and sex. Some outcomes were adjusted further—see tables for details.

#### Health-care usage

Four types of health-care usage were assessed: talking to a doctor, attending the hospital outpatient department, being hospitalised as a day patient, and being hospitalised as an inpatient. In order to compare health-care use with the general population, the 2002 General Household Survey (GHS) served as the general population sample (Richards *et al*, 2004). Multivariable generalised estimating equation (GEE) logistic regression modelling was used to calculate odds ratios (ORs) for health-care usage among bone sarcoma survivors compared to that expected from the general population sample (Rebholz *et al*, 2011).

#### Psychosocial outcomes

The survivors' education level, smoking history, and alcohol consumption were compared with the general population using the 2002 GHS (Richards *et al*, 2004) as the reference sample, whereas marital status was compared with the National Marriage Registry (Office for National Statistics, 2002). Multivariable GEE logistic regression was used to compare educational attainment, smoking status, and alcohol use between survivors and the general population sample (Frobisher *et al*, 2007; Frobisher *et al*, 2008; Frobisher *et al*, 2010; Lancashire *et al*, 2010). ORs were calculated using pooled Mantel–Haenszel tests to compare marital status between survivors and the general population sample.

#### Health status

Version one of the Short Form 36 (SF-36) Health Survey was used to measure self-reported health status by the following eight scales: physical function, role-physical, role-emotional, social functioning, mental health, vitality, pain, and general health perception. External comparisons were conducted using the Oxford Healthy Life Survey (OHLS) as the general population sample. Multivariable linear regression and direct standardisation were used to compare bone sarcoma survivors and the OHLS population.

All analyses were undertaken using Stata 12.1 (StataCorp, College Station, TX, USA). Statistical significance was defined as a two-sided *P*-value<0.05.

## Results

### Cohort characteristics

Of the 664 bone sarcoma survivors, 309 (46.5%) were diagnosed with osteosarcoma, 260 (39.2%) were diagnosed with Ewing sarcoma, 26 (3.9%) were diagnosed with chondrosarcoma, 48 (7.2%) were diagnosed with other specified bone sarcomas (e.g., fibromatous neoplasms, giant cell tumours, chordomas, and miscellaneous bone tumours), and 21 (3.2%) were diagnosed with an unspecified bone sarcoma. The mean age at diagnosis was 10.8 and the average attained age was 39.4 years (Table 1). Osteosarcoma survivors were older at diagnosis and had a higher attained age compared with Ewing sarcoma survivors. Excluding missing information, 60.2% and 60.0% of survivors received radiotherapy and chemotherapy, respectively. In general, characteristics of the 411 survivors who returned a questionnaire were similar both overall and by tumour type to the entire BCCSS bone sarcoma cohort, except that only 3.7% had died by 31 December 2010 subsequent to completing a questionnaire.

### Record linkage ascertained data

#### Cause-specific mortality

Overall, bone sarcoma survivors experienced seven times (SMR: 7.0, 95% confidence interval (CI): 5.9–8.3) the number of deaths expected from the general population with 72 (95% CI: 57.2–85.8) excess deaths per 10 000 person-years (Table 2). The largest excess was for neoplastic-related causes in both relative and absolute terms; recurrences and SPNs accounted for 71.2% and 22.6% of all excess deaths, respectively. When the SMR was assessed by follow-up, a striking difference was observed; the overall SMR was 12.7-times (95% CI: 10.5–15.2) that expected during 0–24 years follow-up and only 1.7-times (95% CI:1.0–2.7) that expected beyond 25 years. Notably, there was an 8-fold decrease in SMRs from 0–24 years to beyond 25 years follow-up for SPN-related deaths. Compared with the general population, the SMR for all-causes was significantly higher (*P*<0.001) for Ewing sarcoma survivors, who had approximately double the SMR and AER of osteosarcoma survivors. Although recurrence and SPN-related deaths accounted for ∼93% of all excess deaths in both tumour types, there was heterogeneity in the proportion of recurrence and SPN excess deaths; recurrences accounted for 59.0% and 80.2% of excess deaths in osteosarcoma and Ewing sarcoma survivors, respectively, whilst the corresponding excess SPN deaths were 34.7% and 13.1%.

**Figure 1:**
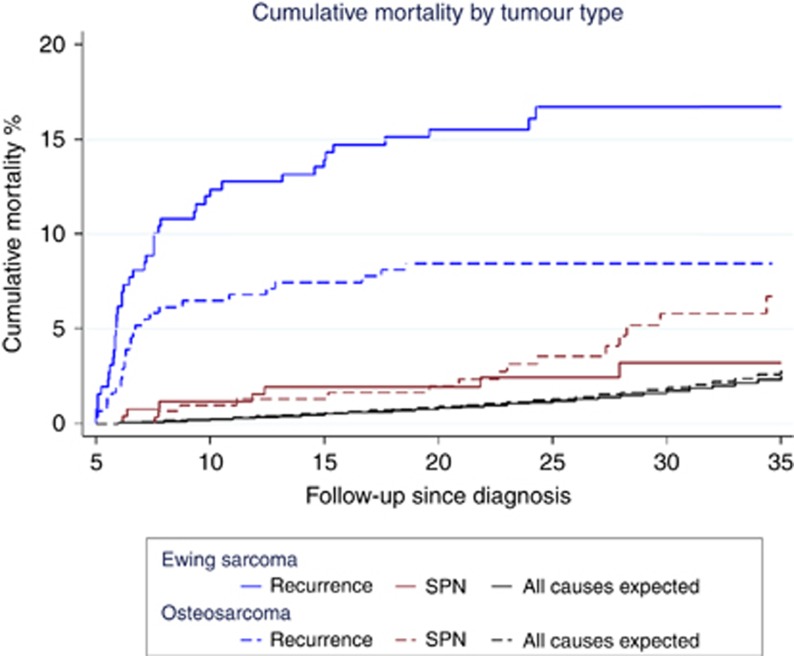
Cumulative mortality of recurrence and second primary neoplasms among childhood bone sarcoma survivors within the British Childhood Cancer Survivor Study (BCCSS) by tumour type.

There was a steep increase in mortality during the initial 5 years following 5-year survival where the cumulative mortality reached 10.4% (95% CI: 8.3–13.0) (Supplementary Figure 1). Subsequently, there was a more gradual incline to 20.6% (95% CI: 17.3–24.3) at 35 years post diagnosis. When stratified by tumour type (Figure 1) a significant difference (*P*=0.004) in cumulative mortality was observed for recurrences, where Ewing sarcoma survivors had nearly double the cumulative mortality at 35 years post diagnosis (osteosarcoma: 8.5% *vs* Ewing sarcoma: 16.7%). Conversely, the cumulative mortality due to SPNs was twice as high for osteosarcoma compared with Ewing sarcoma survivors at the same point (osteosarcoma: 6.7% *vs* Ewing sarcoma: 3.2%).

#### Subsequent primary neoplasms

Bone sarcoma survivors were 4.4-times (95% CI: 3.3–5.8) more likely to experience a SPN than expected and had 29.3 (95% CI: 18.7–39.9) excess SPNs per 10 000 person-years (Table 3). By SPN cancer type, survivors overall and by tumour type were at a considerably higher risk of developing a subsequent bone neoplasm and to a lesser extent a breast neoplasm. Specifically overall, survivors were 136.3- (95% CI: 79.2–234.8) and 4.5-times (95% CI: 2.6–8.0) more at risk than the general population for bone and breast cancers, respectively. When the SIRs were assessed by follow-up, a 8.4-fold (95% CI: 6.1–11.2) increased risk was observed during 0–24 years, where the SIR for subsequent breast and bone cancer were 10.8 (95% CI: 5.2–19.9) and 154.3 (95% CI: 82.2–263.8), respectively. Beyond 25 years of follow-up, the SIR for any SPN was not significantly higher in survivors than expected from the general population.

There was a continuous and steady increase in cumulative incidence for SPNs over follow-up, ultimately reaching 8.3% (95% CI: 5.9–11.2) at 30 years post diagnosis (Supplementary Figure 2). When stratified by tumour type, the cumulative incidence curves were nearly identical to each other and to bone sarcoma survivors overall (*P*>0.05).

### Questionnaire ascertained data

#### Health-care usage

Compared with the general population sample, bone sarcoma survivors were almost three times (OR: 2.9, 95% CI: 2.3–3.7) more likely to have visited an outpatient hospital department in the previous three months (Table 4). Survivors were also over twice (OR: 2.4, 95% CI: 1.7–3.4) more likely to be hospitalised as an inpatient during the previous year than the general population sample. When analysed by tumour type, both osteosarcoma and Ewing sarcoma survivors had significantly higher odds of attending the hospital as an outpatient or inpatient than expected.

#### Psychosocial outcomes

Bone sarcoma survivors overall were comparable to the general population sample for being ever-married, a current drinker, or consuming harmful amounts of alcohol (Table 4). Survivors were, however, significantly less likely to be a current smoker (OR: 0.6, 95% CI: 0.5–0.8) and consume alcohol over recommendations (OR: 0.7, 95% CI: 0.5–0.9) than the general population sample. Compared to that expected, survivors performed well in obtaining educational qualifications and were 70% more likely to have obtained at least O-levels (OR: 1.7, 95% CI: 1.3–2.1). When analysed by tumour type, the odds for each psychosocial outcome were comparable to the overall finding.

#### Health status

**Figure 2:**
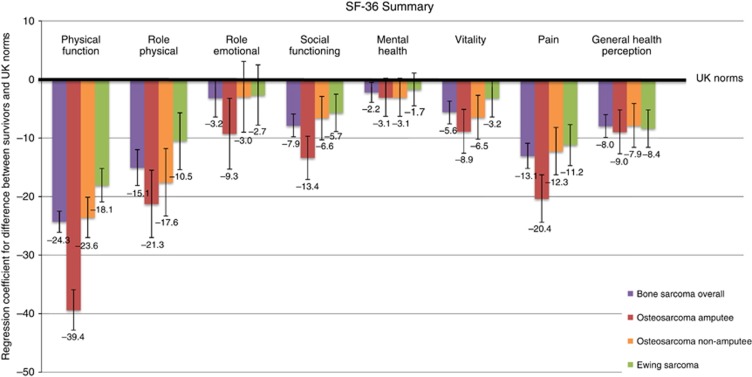
Sex and age adjusted regression coefficients and corresponding 95% confidence intervals for differences in SF-36 health status scales between bone sarcoma, osteosarcoma amputees, osteosarcoma non-amputees, and Ewing sarcoma survivors *vs* UK norms.

Compared with the general population sample, bone sarcoma survivors overall were significantly more limited in all SF-36 scales with the exception of role-emotional (Figure 2). The most notable differences occurred in physical function, role-physical, and pain. For the individual components of the physical function scale (Supplementary Figure 3), 54% and 61% of survivors were limited in ‘moderate activities' and ‘walking more than one mile' compared with the 8% and 11% expected from the general population sample, respectively. In the role-physical scale (Supplementary Figure 4), the largest difference between the survivors and general population sample was in ‘being limited in the kind of work and activities', although all component questions reported at least a 10% deficit. Finally, for the pain scale (Supplementary Figure 5), survivors reported more bodily pain (12% *vs* 5%) and more pain interference (16% *vs* 5%) during the past 4 weeks compared with the general population sample.

When stratified by tumour type, osteosarcoma survivors were assessed additionally by amputation status, where only arm or leg amputations as a form of initial treatment for the first primary tumour were included. Compared with the general population sample, osteosarcoma amputee survivors reported being the most limited in all scales relative to osteosarcoma non-amputees and Ewing sarcoma survivors, with a significantly (*P*<0.001) higher disadvantage in physical function (Figure 2).

## Discussion

This is the first large-scale population-based study to provide a comprehensive description of long-term health and social outcomes among a large cohort of 5-year bone sarcoma survivors, both overall and by tumour type, beyond 35 years post diagnosis. Mortality estimates in this cohort were elevated seven times that expected and varied significantly between tumour types, which were consistent with previous findings of a large-scale US study (Armstrong *et al*, 2009). Past studies have also shown the principal cause of death was neoplastic-related (MacArthur *et al*, 2007; Ginsberg *et al*, 2010; Nagarajan *et al*, 2011). However, to our knowledge, this is the first study that has shown substantial differences when comparing excess and cumulative mortality between tumour types; osteosarcoma survivors had double the cumulative mortality for SPNs compared with Ewing sarcoma and Ewing sarcoma survivors had double the cumulative mortality for recurrences compared with osteosarcoma at 35 years post diagnosis. The osteosarcoma survivors in this study were much more likely to have an amputation than Ewing sarcoma, which may partially explain why osteosarcoma survivors were less likely to have recurred (Grimer *et al*, 2002). Additionally, due to the extended follow-up available, this is the first study to show that beyond 25 years follow-up the risk of dying from all-causes is comparable to the general population and unlikely to exceed 2.7-fold that expected. This is in contrast to dying before 25 years of follow-up, where the risk is 12.7-fold that expected. This provides important evidence for clinicians who monitor survivors treated in similar decades to those included in the BCCSS. A possible explanation for this striking absence or low risk of excess mortality with extended follow-up may relate to our previous observation that, as the overall cohort of childhood cancer survivors ages, a large proportion of excess deaths are attributed to SPNs (Reulen *et al*, 2010), particularly breast, digestive, genitourinary, and lung carcinomas. Although carcinomas of these sites are common cancers of adulthood in the general population, in childhood cancer survivors they are principally caused by direct radiotherapy exposure (Reulen *et al*, 2011). As 80% of the bone sarcomas included here were diagnosed in the limb, there is unlikely to have been much direct exposure from radiotherapy to tissues of these sites due to the lack of proximity of the radiotherapy fields.

The overall and tumour type-specific SIRs for SPNs were consistent with previous studies (Magnani *et al*, 1996; Cardous-Ubbink *et al*, 2007; Inskip and Curtis, 2007; Friedman *et al*, 2010). Additionally, our findings are consistent with previous literature in that the most common SPN was breast cancer for osteosarcoma survivors and bone cancer for Ewing sarcoma survivors (Inskip and Curtis, 2007). Due to the extended follow-up available, this is the first study to show that the risk of developing SPNs was 9.9-fold the expected during 0–24 years follow-up and comparable to the general population beyond 25 years follow-up, where it was unlikely to exceed 2.0-fold that expected. Notably, all thirteen bone cancers occurred before 25 years follow-up, nine of which developed inside or on the edge of tissue directly irradiated to treat the original bone sarcoma and one in a survivor diagnosed with a p53 mutation. This corresponds with our previous work that found that bone cancer is the most common SPN after a first primary bone sarcoma (Reulen *et al*, 2011), which is principally attributable to exposure of the SPN site to radiation during treatment for the first cancer (Tucker *et al*, 1987; Hawkins *et al*, 1996; Schwartz *et al*, 2014). Of the two breast cancers observed subsequent to 25 years follow-up, both developed in survivors previously treated for a bone sarcoma of a lower limb with unknown p53 status.

Consistent with other studies (Eiser *et al*, 2001; Hudson *et al*, 2003; Zeltzer *et al*, 2008), we reported that survivors were severely limited in health status, in particular physical function and pain. Although previous studies have suggested that health status among amputees is generally similar to non-amputees (Eiser and Grimer, 1999; Nagarajan *et al*, 2004; Paul, 2008; Eiser, 2009; Nagarajan *et al*, 2009; Barrera *et al*, 2012), we found that osteosarcoma amputees reported the worst health status for all scales, with significantly higher limitations in physical function relative to osteosarcoma non-amputees and Ewing sarcoma (95% of which were non-amputees) survivors.

Although we report here on appreciable proportions of the bone sarcoma survivors experiencing detrimental effects to their health, many of their social outcomes were favourable. In fact, our findings suggest that survivors were more likely than expected to obtain some types of educational qualification and less likely to be a current smoker than expected from the general population.

### Current guidelines and recommendations

The Bone Cancer Research Trust currently recommends yearly follow-up after 5-year survival (Newby and Unsworth, 2013a, 2013b). From the evidence presented here, 74% and 21% of all excess deaths before 25 years of follow-up were due to recurrence and SPN, respectively, and therefore monitoring of survivors for recurrences and SPNs, particularly during the period 5–10 years post diagnosis where risk of recurrence is highest, could help prevent premature mortality. Bone and breast SPNs should also be a particular concern and regular follow-up should be provided, particularly in the period 0–24 years following 5-year survival for bone SPNs. Factsheets given to childhood cancer survivors could further expand upon the risk of recurrence and SPNs and the potential for early diagnosis by detailing more precisely signs and symptoms relating to bone and breast neoplasms. Furthermore, the substantial excess risks of specific physical limitations and pain are likely to be useful for risk stratification and possible interventions that seek to reduce morbidity and the practical difficulties that survivors may face.

### Limitations

Although the findings in this paper may not be generalisable for children diagnosed after 1991, the purpose of this study was to address the long-term, beyond 35 years post diagnosis, outcomes that childhood bone sarcoma survivors are currently facing. We acknowledge reassessment is necessary and recommend further analyses to be conducted on the recently extended BCCSS cohort, which includes 5-year survivors diagnosed from 1992 to 2006. Furthermore, as a large proportion of bone sarcoma diagnoses occur in individuals aged over 14 years, we recommend further analyses on adverse outcomes to be assessed using the Teenage and Young Adult Cancer Survivor Study (TYACSS), which we have established recently and includes all 5-year survivors diagnosed from age 15–39 in England and Wales between 1970 and 2006. Finally, a potential limitation of our study is the lack of detailed treatment information. Although we report a large reduction in excess mortality and SPNs beyond 25 years follow-up, those followed-up for <25 years are more likely to be treated differently due to the introduction of chemotherapy. Thus, reassessment of these more recently diagnosed individuals is essential in order to determine whether the decreases in risk reported in this study remain with newer treatment practices. Nevertheless, due to our population-based design, the evidence presented here provides a reliable and unbiased basis to update clinical follow-up guidelines in relation to bone sarcoma survivors diagnosed before age 15 and treated before 1992 in Great Britain by using cancer diagnosis as a proxy.

## Conclusions

In conclusion, childhood bone sarcoma survivors diagnosed between 1940 and 1991 in this cohort are at substantial risk of death and SPNs up to 25 years after 5-year survival, but the risk is greatly reduced thereafter. Survivors additionally face difficulties in daily life due to their excess prevalence of poor physical health status. As there are variations in the degree of excess depending on the specific outcome and whether they survived osteosarcoma or Ewing sarcoma, risk needs to be assessed in a stratified way. These findings should provide useful evidence for risk stratification, updating clinical follow-up guidelines, and possible intervention studies.

## Figures and Tables

**Table 1:** Characteristics of bone sarcoma study population overall and by tumour type

	**Available survivors for data linkage (*****N*****=664)**			**Available questionnaire completed survivors (*****N*****=411)**		
**Characteristic**	**All** ***N*** **(%)**	**Osteosarcoma** ***n*** **(%)**	**Ewing sarcoma** ***n*** **(%)**	**All** ***N*** **(%)**	**Osteosarcoma** ***n*** **(%)**	**Ewing sarcoma** ***n*** **(%)**
**Sex**						
Male	345 (52.0)	150 (48.5)	138 (53.1)	200 (48.7)	96 (47.8)	74 (48.1)
Female	319 (48.0)	159 (51.5)	122 (46.9)	211 (51.3)	105 (52.2)	80 (52.0)
**Cancer site**						
Upper limbs	92 (13.9)	34 (11.0)	48 (18.5)	56 (13.7)	21 (10.5)	32 (20.8)
Lower limbs	436 (65.7)	262 (84.8)	132 (50.8)	273 (66.5)	170 (84.6)	75 (48.7)
Bones of skull and face	42 (6.4)	5 (1.6)	10 (3.9)	26 (6.3)	4 (2.0)	6 (3.9)
Vertebral column	24 (3.6)	3 (1.0)	15 (5.8)	13 (3.2)	3 (1.5)	7 (4.6)
Rib, sternum, clavicle	28 (4.2)	1 (0.3)	25 (9.6)	20 (4.9)	1 (0.5)	17 (11.0)
Pelvic, sacrum, coccyx	33 (5.0)	2 (0.7)	27 (10.4)	19 (4.6)	2 (1.0)	15 (9.7)
Other	9 (1.4)	2 (0.7)	3 (1.2)	4 (1.0)	0 (0)	2 (1.3)
**Age at diagnosis**						
Mean (range)	10.8 (0.1–15.0)	11.5 (2.3–15.0)	10.2 (1.5–15.0)	10.8 (1.3–15.0)	11.6 (3.2–15.0)	10.0 (2.0–15.0)
0–4 years	40 (6.0)	8 (2.6)	22 (8.5)	22 (5.4)	4 (2.0)	13 (8.4)
5–9 years	185 (27.9)	74 (24.0)	84 (32.3)	122 (29.7)	46 (22.9)	58 (37.7)
10–14 years	439 (66.1)	227 (73.5)	154 (59.2)	267 (65.0)	151 (75.1)	83 (53.9)
**Attained age at exit**						
Mean (range)	39.4 (7.5–76.8)^a^	40.9 (10.0–71.9)^a^	35.7 (7.5–65.2)^a^	43.3 (22.4–76.8)^b^	44.6 (22.9–71.9)^b^	39.4 (22.4–65.2)^b^
16–24 years	89 (13.4)	31 (10.0)	47 (18.1)	71 (17.3)	23 (11.4)	43 (27.9)
25–34 years	155 (23.3)	62 (20.1)	78 (30.0)	169 (41.1)	83 (41.3)	72 (46.8)
35–44 years	218 (32.8)	108 (35.0)	90 (34.6)	92 (22.4)	53 (26.4)	25 (16.2)
45+ years	202 (30.4)	108 (35.0)	45 (17.3)	79 (19.2)	42 (20.9)	14 (9.1)
**Radiotherapy**^c^						
No	201 (39.8)	150 (59.3)	17 (9.9)	125 (40.3)	98 (58.3)	10 (10.5)
Yes	304 (60.2)	103 (40.7)	154 (90.1)	185 (59.7)	70 (41.7)	85 (89.5)
**Chemotherapy**^c^						
No	195 (40.0)	93 (37.5)	40 (23.8)	109 (36.7)	58 (35.6)	16 (17.2)
Yes	292 (60.0)	155 (62.5)	128 (76.2)	188 (63.3)	105 (64.4)	77 (82.8)
**Surgery**^c^						
No	160 (31.3)	34 (13.2)	109 (63.4)	93 (29.4)	19 (11.1)	62 (64.6)
Yes	352 (68.8)	223 (86.8)	63 (36.6)	223 (70.6)	152 (88.9)	34 (35.4)
**Vital status**^a^						
Alive	533 (80.3)	256 (82.9)	203 (78.1)	396 (96.4)	193 (96.0)	150 (97.4)
Dead	131 (19.7)	53 (17.2)	57 (21.9)	15 (3.7)	8 (4.0)	4 (2.6)

a Age at 31 December 2010 or death/embarkation (if before 31 December 2010)—relevant to the mortality analyses.

b Age at questionnaire completion—relevant to outcomes measured by the questionnaire.

c Missing data: radiotherapy (all data linkage)=139, radiotherapy (all questionnaire)=101; chemotherapy (all data linkage)=157, chemotherapy (all questionnaire)=114; surgery (all data linkage)=152, Surgery (all questionnaire)=95.

**Table 2:** All-cause and cause-specific^a^ standardised mortality ratios and absolute excess risk for bone sarcoma survivors within the British Childhood Cancer Survivor Study (BCCSS) overall and by tumour type

	**All bone sarcoma survivors**										**Osteosarcoma survivors**				**Ewing sarcoma survivors**				
	**Overall**				**<25 Years follow-up**^b^			**⩾25 Years follow-up**^b^			**Overall**				**Overall**				
**Underlying cause of death**	**Person-years**	***O*****/*****E***	**SMR (95% CI)**	**AER (95% CI)**^c^	***O*****/*****E***	**SMR (95% CI)**	**AER (95% CI)**^c^	***O*****/*****E***	**SMR (95% CI)**	**AER (95% CI)**^c^	**Person-years**	***O*****/*****E***	**SMR (95% CI)**	**AER (95% CI)**^c^	**Person-years**	***O*****/*****E***	**SMR (95% CI)**	**AER (95% CI)**^c^	***P*****-value**^d^
All-causes	15 678	131/18.7	7.0 (5.9, 8.3)	71.6 (57.3, 86.0)	115/9.1	12.7 (10.5, 15.2)	82.7 (66.3, 99.1)	16/9.6	1.7 (1.0,2.7)	22.3 (−5.0,49.7)	7539	53/8.9	6.0 (4.5, 7.8)	58.5 (39.6, 77.5)	5327	57/4.6	12.3 (9.4, 16.0)	98.3 (70.6, 126.1)	<0.001
Recurrence	15 678	80/0.0	NA	51.0 (39.8, 62.2)	79/0.0	NA	61.6 (48.1,75.2)	1/0.0	NA	3.5 (−3.4, 10.3)	7539	26/0.0	NA	34.5 (21.2, 47.7)	5327	42/0.0	NA	78.8 (55.0, 102.7)	
SPN	15 678	31/5.5	5.6 (3.8, 8.0)	16.2 (9.3, 23.2)	24/1.7	14.4 (9.2, 21.5)	17.4 (9.9, 24.9)	7/3.9	1.8 (0.7, 3.7)	10.9 (−7.2, 29.0)	7539	18/2.7	6.7 (4.0, 10.6)	20.3 (9.3, 31.3)	5327	8/1.1	7.1 (3.1, 14.1)	12.9 (2.5, 23.3)	0.772
Circulatory	15 678	8/3.6	2.3 (1.0, 4.4)	2.8 (−0.7, 6.4)	3/1.0	3.0 (0.6, 8.7)	1.6 (−1.1, 4.2)	5/2.5	2.0 (0.6, 4.6)	8.6 (−6.7, 23.9)	7539	3/1.7	1.8 (0.4, 5.3)	1.8 (−2.7, 6.3)	5327	2/0.7	3.0 (0.4, 11.0)	2.5 (−2.7, 7.7)	0.598
External	15 678	5/4.7	1.1 (0.3, 2.5)	0.2 (−2.6, 3.0)	5/3.9	1.3 (0.4, 3.0)	0.8 (−2.6, 4.3)	0/−	NP	NP	7539	3/2.2	1.4 (0.3, 4.0)	1.0 (−3.5, 5.5)	5327	1/1.6	0.6 (0.0, 3.6)	−1.0 (−4.7, 2.6)	0.535

Abbreviations: AER=absolute excess risk; CI=confidence intervals; *E*=expected number; NA=not applicable; NP=not possible to reliably calculate due to very small expected number; *O*=observed number; SMR=standardised mortality ratios.

a Results are only reported for underlying causes of deaths with at least five observed events overall. Other causes of death were: four genitourinary, one digestive, one infection, and one unknown.

b From five-year survival.

c Per 10 000 person-years.

d Comparing SMRs for osteosarcoma and Ewing sarcoma survivors.

SMRs and AERs where there are <5 observed events should be interpreted with caution.

**Table 3:** Overall and site-specific^a^ standardised incidence ratios and absolute excess risks of second primary neoplasms for bone sarcoma survivors within the British Childhood Cancer Survivor Study (BCCSS)

	**All bone sarcoma survivors**															
	**Overall**			**<25 Years follow-up**^b^			**⩾25 Years follow-up**^b^			**Osteosarcoma survivors**			**Ewing sarcoma survivors**			
**SPN**	***O*****/*****E***	**SIR (95% CI)**	**AER (95% CI)**^c^	***O*****/*****E***	**SIR (95% CI)**	**AER (95% CI)**^c^	***O*****/*****E***	**SIR (95% CI)**	**AER (95% CI)**^c^	***O*****/*****E***	**SIR (95% CI)**	**AER (95% CI)**^c^	***O*****/*****E***	**SIR (95% CI)**	**AER (95% CI)**^c^	***P-*****value**^d^
Any cancer site	49/11.2	4.4 (3.3, 5.8)	29.3 (18.7, 39.9)	44/5.3	8.4 (6.1, 11.2)	34.7 (23.1, 46.3)	5/5.9	0.8 (0.3, 2.0)	−5.1 (−30.2, 20.0)	23/6.0	3.9 (2.6, 5.8)	26.1 (11.7, 40.5)	19/2.8	6.7 (5.3, 10.6)	35.7 (16.9, 54.6)	0.070
Breast	12/2.6	4.5 (2.6, 8.0)	7.2 (2.0, 12.5)	10/0.9	10.8 (5.2, 19.9)	8.1 (2.6, 13.7)	2/1.7	1.2 (0.1, 4.2)	1.6 (−14.2, 17.5)	6/1.5	4.0 (1.8, 8.8)	6.9 (−0.5, 14.2)	5/0.6	7.8 (3.2, 18.6)	9.6 (−0.1, 19.3)	0.236
Bone	13/0.1	136.3 (79.2, 234.8)	10.0 (4.5, 15.5)	13/0.1	154.3 (82.2, 263.8)	11.6 (5.2, 17.9)	0/−	NP	NP	3/0.05	65.4 (21.1, 202.7)	4.5 (−0.7, 9.7)	8/0.04	223.0 (111.5, 445.9)	17.6 (5.4, 29.8)	0.057

Abbreviations: AER=absolute excess risk; CI=confidence intervals; *E*=expected number; NA=not applicable; NP=not possible to reliably calculate due to very small expected number; *O*=observed number; SIR=standardised incidence ratios; SMR=standardised mortality ratios.

a Results are only reported for site-specific SPNs with at least five observed events overall. Other SPNs were: four genitourinary, four bladder, three digestive, three connective and soft tissue, three malignant neoplasms with unspecified sites, two gliomas, two Hodgkin lymphoma, two NHL, two leukemia, one respiratory, one eye, one thyroid.

b From five-year survival.

c AER is shown per 10 000 person-years.

d Comparing SIRs for osteosarcoma and Ewing sarcoma survivors.

SIRs and AERs where there are <5 observed events should be interpreted with caution.

**Table 4:** Percentages and odds ratios (with corresponding 95% CIs) for the likelihood of use of health services and psychosocial outcomes in bone sarcoma survivors within the British Childhood Cancer Survivor Study (BCCSS) compared with the general population of Britain

	**UK norms (ref)**	**Bone sarcoma overall OR (95% CI)**	**Osteosarcoma OR (95% CI)**	**Ewing sarcoma OR (95% CI)**
**Marital Status**^a^				
Males ever-married	1.0	0.7 (0.5, 1.0)	1.0 (0.6, 1.6)	0.7 (0.4, 1.3)
Females ever-married	1.0	0.8 (0.6, 1.1)	0.8 (0.5, 1.2)	0.9 (0.6, 1.4)
**Education**^b^				
University degree or higher	1.0	1.2 (1.0, 1.6)	1.5 (1.1, 2.1)	1.0 (0.8, 1.5)
Teaching qualification or higher	1.0	1.1 (0.9, 1.4)	1.3 (1.0, 1.7)	1.0 (0.7, 1.4)
A-levels or higher^c^	1.0	1.2 (1.0, 1.5)	1.2 (0.9, 1.6)	1.1 (0.8, 1.5)
O-levels or higher^d^	1.0	1.7 (1.3, 2.1)	1.8 (1.2, 2.6)	2.0 (1.2, 3.1)
**Alcohol**^e^				
Current drinker	1.0	0.8 (0.6, 1.1)	1.0 (0.6, 1.6)	0.8 (0.5, 1.3)
Consuming over recommendations	1.0	0.7 (0.5, 0.9)	0.7 (0.5, 1.1)	0.5 (0.3, 0.9)
Consuming harmful amounts	1.0	0.7 (0.4, 1.1)	0.6 (0.3, 1.4)	0.7 (0.3, 1.5)
**Smoking**^e^				
Current smoker	1.0	0.6 (0.5, 0.8)	0.7 (0.5, 1.0)	0.6 (0.4, 0.9)
**Use of health services**^f^				
Talked to a doctor^g^	1.0	1.2 (0.9, 1.6)	1.3 (0.9, 1.8)	1.2 (0.8, 1.8)
Attended as outpatient^g^	1.0	2.9 (2.3, 3.7)	2.9 (2.1, 4.0)	3.2 (2.2, 4.7)
Attended as day patient^h^	1.0	1.1 (0.7, 1.5)	1.2 (0.7, 1.9)	1.0 (0.6, 1.8)
Attended as inpatient^h^	1.0	2.4 (1.7, 3.4)	2.5 (1.6, 3.9)	2.8 (1.7, 4.7)

Abbreviations: OR=odds ratio; CI=confidence interval.

a From a pooled Mantel–Haenszel model controlling for attained age.

b From a GEE multivariable logistic regression controlling for age at questionnaire completion and sex (taking into account the GHS weighting factor).

c Degree received at age 16.

d Degree received at age 18.

e From a GEE multivariable logistic model adjusting for attained age (⩽69 years), sex, marital status, socioeconomic classification, educational attainment, and region (taking into account the GHS weighting factor).

f From a GEE multivariable logistic model adjusting for age at questionnaire completion, sex, and educational attainment.

g Excluding women who were pregnant at time of survey.

h Excluding visits for having a baby.
