# Supplementary material for: Long-term adverse outcomes in survivors of childhood bone sarcoma: the British Childhood Cancer Survivor Study
Source: Br J Cancer. 2015 May 19;112(12):1857–65. doi: 10.1038/bjc.2015.159 (PMC4580396; doi:10.1038/bjc.2015.159)
Supplement: Supplementary Information [file bjc2015159x1.doc]

1. **ONLINE ONLY:** Figure 1: Cumulative mortality of neoplastic versus non-neoplastic causes of death among childhood bone sarcoma survivors within the British Childhood Cancer Survivor Study (BCCSS)


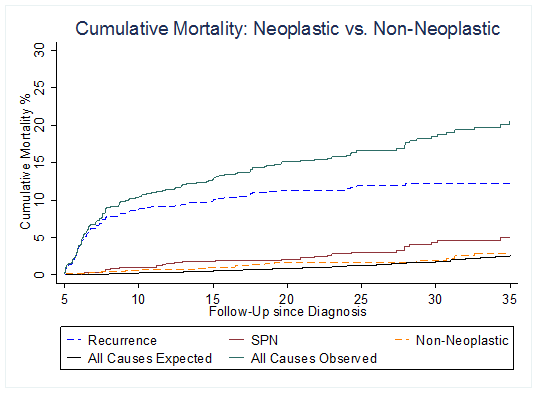


2. **ONLINE ONLY:** Figure 2: Observed cumulative incidence of a subsequent primary neoplasm among bone sarcoma survivors within the British Childhood Cancer Survivor Study (BCCSS) overall and by tumor type

3. **ONLINE ONLY:** Figure 3: Directly (age and sex) standardized prevalence and corresponding 95% confidence intervals, of reporting being limited† in specific daily activity or other problem related to physical function score of SF-36


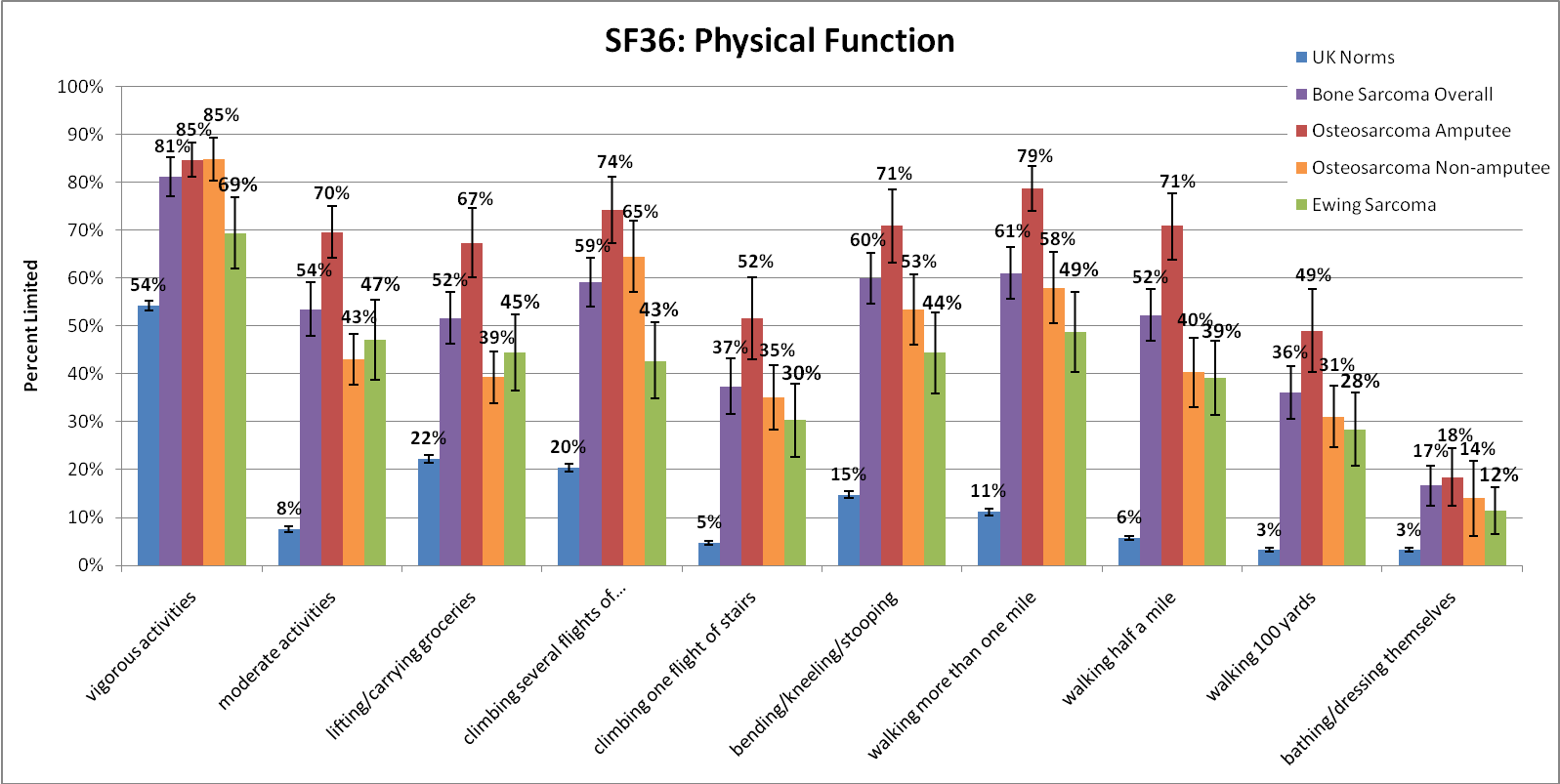


†Limited defined as reporting “yes, limited a lot” or “yes, limited a little” for relevant question of BCCSS questionnaire

4. **ONLINE ONLY:** Figure 4: Directly (age and sex) standardized prevalence and corresponding 95% confidence intervals, of reporting being limited† in specific daily activity or other problem related to role limitation physical score of SF-36


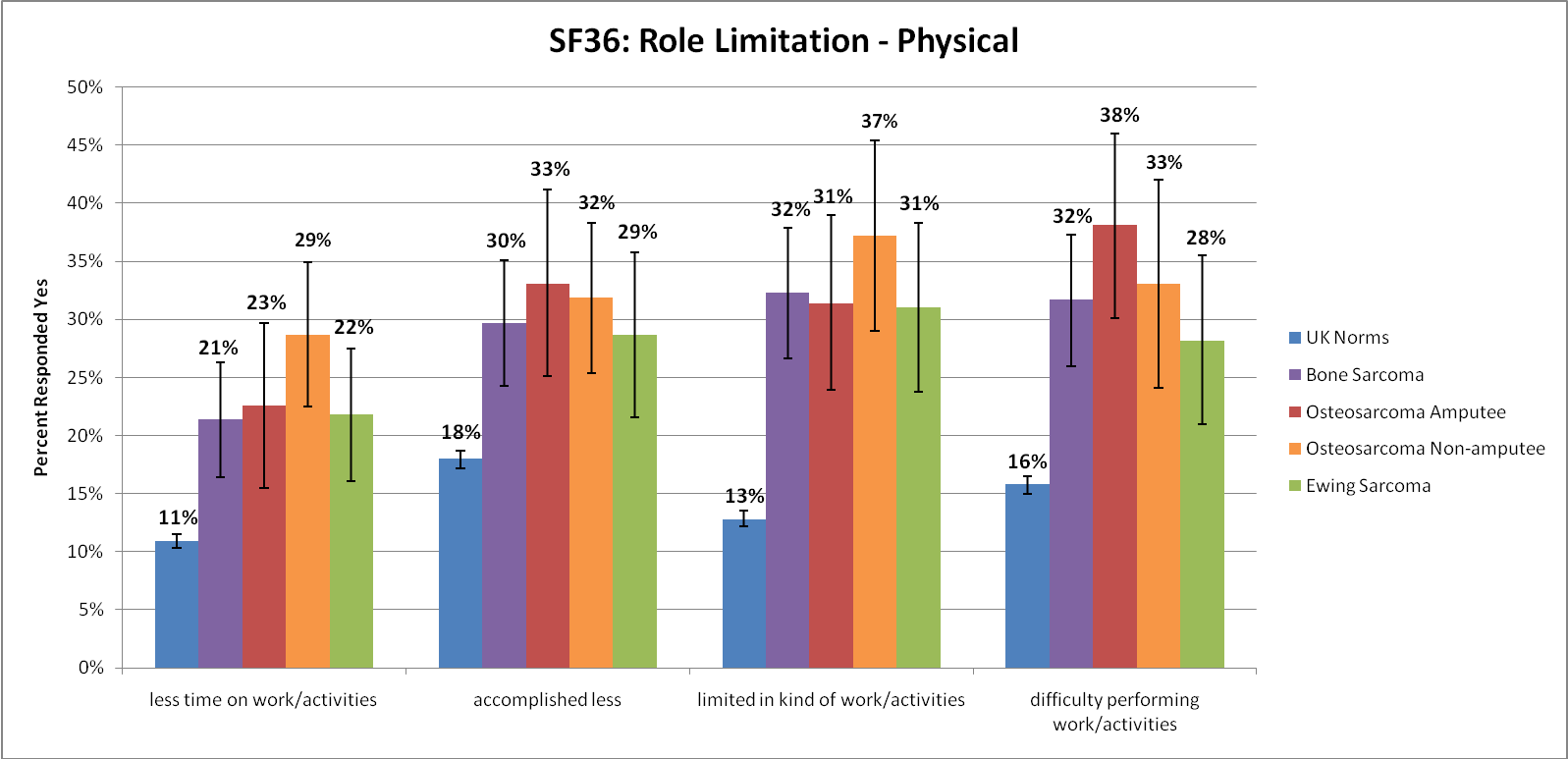


†Limited defined as reporting “yes” for relevant question of BCCSS questionnaire

5. **ONLINE ONLY:** Figure 5: Directly (age and sex) standardized prevalence and corresponding 95% confidence intervals, of reporting being limited† in specific daily activity or other problem related to pain score of SF-36


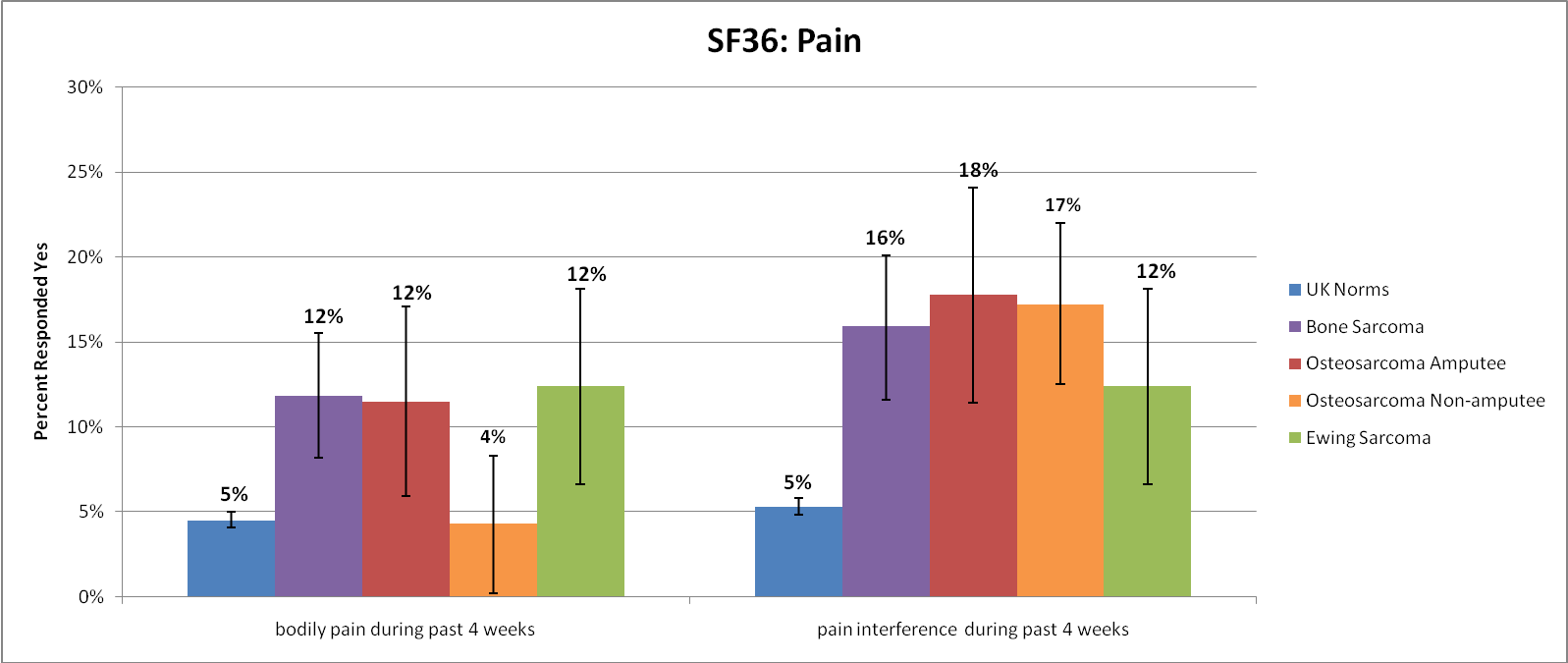


†Limited defined as reporting “severe/very severe” or “quite a bit/ extremely” for relevant question of BCCSS questionnaire
